# Patient perspectives on variant reclassification after cancer susceptibility testing

**DOI:** 10.1002/mgg3.1275

**Published:** 2020-04-24

**Authors:** Colin M. E. Halverson, Laurie M. Connors, Bronson C. Wessinger, Ellen W. Clayton, Georgia L. Wiesner

**Affiliations:** ^1^ Center for Bioethics Indiana University School of Medicine Indianapolis IN USA; ^2^ Regenstrief Institute Indianapolis IN USA; ^3^ School of Nursing Vanderbilt University Nashville TN USA; ^4^ Vanderbilt University School of Medicine Nashville TN USA; ^5^ Center for Biomedical Ethics and Society Vanderbilt University Medical Center Nashville TN USA; ^6^ Department of Pediatrics Vanderbilt University Medical Center Nashville TN USA; ^7^ School of Law Vanderbilt University Nashville TN USA; ^8^ Department of Medicine Vanderbilt University Medical Center Nashville TN USA; ^9^ Vanderbilt‐Ingram Cancer Center Vanderbilt University Medical Center Nashville TN USA

**Keywords:** ethics, genetics, reclassification, recontact, uncertainty

## Abstract

**Background:**

Little is known about the impact of reclassification on patients’ perception of medical uncertainty or trust in genetics‐based clinical care.

**Methods:**

Semistructured telephone interviews were conducted with 20 patients who had received a reclassified genetic test result related to hereditary cancer. All participants had undergone genetic counseling and testing for cancer susceptibility at Vanderbilt‐Ingram Cancer Center Hereditary Cancer Clinic within the last six years.

**Results:**

Most of the participants did not express distress related to the variant reclassification and only a minority expressed a decrease in trust in medical genetics. However, recall of the new interpretation was limited, even though all participants were recontacted by letter, phone, or clinic visit.

**Conclusion:**

Reclassification of genetic tests is an important issue in modern healthcare because changes in interpretation have the potential to alter previously recommended management. Participants in this study did not express strong feelings of mistrust or doubt about their genetic evaluation. However, there was a low level of comprehension and information retention related to the updated report. Future research can build on this study to improve communication with patients about their reclassified results.

## Graphical abstract

Little is known about the impact of reclassification on patients’ perception of medical uncertainty or trust in genetics‐based clinical care. Participants in this study did not express strong feelings of mistrust or doubt about their genetic evaluation. However, there was a low level of comprehension and information retention related to the updated report.

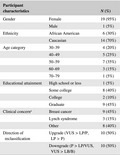

## 1 INTRODUCTION

Genetic testing for diagnosing and predicting disease has become a mainstay of genetic medicine. The field is still young, and our knowledge of the relationship between genotype and human health is constantly evolving, such that understandings of the clinical impact a patient's test result may change over time. Because of this, testing laboratories have developed internal protocols for variant interpretation, reinterpretation, and communication of results with the ordering health care provider (Murray, Cerrato, Bennett, & Jarvik, 2011).

While there are many competing classification systems for annotating genetic variants (Halverson, 2019;Shoenbill, Fost, Tachinardi, & Mendonca, 2014), the American College of Medical Genetics and Genomics and the Association for Molecular Pathology recommend a five‐tier classification system based on the variants’ assumed pathogenicity (Richards et al., 2015). Interpretations of a pathogenic or likely pathogenic variant in genes linked to human cancers may be clinically actionable, meaning that clinicians can implement specific measures to treat or prevent the development of the disorder (Turner, Rao, Morgan, Vnencak‐Jones, & Wiesner, 2019). Furthermore, cascade testing of family members is usually recommended for pathogenic or likely pathogenic variants (Slavin, Manjarrez, Pritchard, Gray, & Weitzel, 2019).

Although all variant interpretations have the potential to change as more is learned, we (Turner et al., 2019) and others (Mersch et al., 2018;Slavin et al., 2019) have shown that reclassification is not uncommon and can significantly alter the management of patients and their families. This is complicated by the fact that there are differences among laboratories in their interpretations of specific genetic variants (Balmaña et al., 2016). Thus, discrepant classifications can be the result of broadly accepted shifts in interpretations of genetic data or of individual laboratories’ differential access to or acceptance of available research.

Most reclassifications take the form of the downgrading of variants of uncertain significance (VUSs) to the status of likely benign or benign, although it is possible that a pathogenic variant can be downgraded to the status of likely benign or VUS as well (Mersch et al., 2018). The reinterpretation can indicate that the patient no longer meets the criteria for specific management guidelines based on the current understandings of the data (Mersch et al., 2018;Turner et al., 2019). Cascade testing might not be recommended for a downgraded variant, which then complicates the management of family members. Upgrading of VUSs also occurs and has more obvious effects on care, making the patient eligible for interventions that previously were not likely recommended.

Reclassification represents an added layer of uncertainty for both practitioners’ and patients’ understanding of genetic information. Uncertainty in clinical knowledge has been linked to patients’ lack of trust in expert practice (Hong & You, 2016;Makhnoon, Garrett, Burke, Bowen, & Shirts, 2019). As has been reported regarding the disclosure of VUSs (Makhnoon et al., 2019;O’Neill et al., 2009), reclassification has the potential to have negative effects on patient attitudes, including trust, anxiety, and understanding and to exacerbate intolerance for uncertainty among patients, thus leading to greater distress.

Little is known about patients’ experience with and understanding of variant reclassification. A study that enrolled family members for the purpose of resolving VUSs showed mixed reactions to reclassifications based on the type of reclassification the patients received (Tsai et al., 2019). The same study also found that the disclosure of uncertain results led many participants to feel frustrated and to distrust their providers (Makhnoon et al., 2019). Far fewer studies have assessed patients’ responses to the reclassification of genetic results (Sexton, Rawlings, McKavanagh, Simons, & Winship, 2015;Wong et al., 2019). Yet an understanding of the reclassification is particularly important for patients with VUSs, as such variants, by definition, are inconclusive and are not meant to inform clinical decision making. However, both patients (Vos et al., 2008) and clinicians (McCullum, Bottorff, Kelly, Kieffer, & Balneaves, 2007;Vos et al., 2008) often inappropriately rely on these results to make decisions about surgery and screening that are inconsistent with current guidelines for management (Eccles, Copson, Maishman, Abraham, & Eccles, 2015;Macklin, Jackson, Atwal, & Hines, 2019;Mersch et al., 2018). In some cases, patients opt for irreversible surgery prior to the downgrading of a VUS to the status of a benign variant (Garcia, Lyon, Littell, & Powell, 2014). These prior studies are important when taken together because they suggest that patients are making serious decisions about their healthcare based on potentially insufficient data, and reclassification most often reveals that the intervention they have chosen to undergo is no longer clinically indicated.

Our limited understanding of patient responses to reclassification is therefore concerning, leading us to wonder whether variant reclassification could adversely impact a patient's trust in medical genetics and in health care more broadly. In order to address this knowledge gap, we conducted a qualitative interview study of patients at Vanderbilt‐Ingram Cancer Center who had received a reclassified genetic test result after previous testing for cancer susceptibility.

## 2 MATERIALS AND METHODS

### 2.1 Participants

We conducted a qualitative study using semistructured interviews with patients evaluated and tested in the Vanderbilt‐Ingram Cancer Center's Hereditary Cancer Clinic within the last six years and who had received amended reports with a reclassification of one or more genetic variants identified on the initial laboratory report. The method of communication followed clinic policy, where patients of clinically significant upgraded or downgraded results were called and then sent a follow‐up letter with a copy of the updated laboratory report. Patients with a downgraded result that did not change current management practices (such as a VUS to LB) were notified by letter or patient portal. Participants were identified through a retrospective review of an IRB‐approved, HIPAA‐compliant Hereditary Cancer REDCap database (Turner et al., 2019) and invited to join the study by letter and follow‐up phone call. REDCap is a Vanderbilt‐developed, web‐based application used to manage online surveys and databases (Harris et al., 2009, 2019). Interviews were conducted over a 1‐year period, between April 2018 and April 2019. This study received approval from Vanderbilt University Medical Center's Institutional Review Board.

We sought to interview 20 patients who were 18 years of age or older and had valid contact information. Our sampling method was purposive, attempting to recruit as many participants as possible whose results could have changed the clinical relevance of the variant or altered clinical management (Turner et al., 2019). As stated, candidates were first sent an invitation letter, notifying them of the project and a phone number to call if they were interested in participating. Two weeks later, researchers began inviting these candidates by phone to enroll in the study. Contact by phone was attempted with candidates up to three times. Those who wished to participate completed an oral consent and scheduled a mutually acceptable time for the phone interview. A separate REDCap database was created with participants’ contact information, clinical data, and genetic test results. Participants were offered a $25 online gift certificate in appreciation for their time.

### 2.2 Interview guide

We developed an in‐depth, semistructured interview guide, which is available in the Data Supplement. In consultation with experienced methodologists and qualitative researchers at Vanderbilt University, our team developed the guide iteratively, designed based on a review of literature related to issues of reclassification, the return of results, and clinical uncertainty.

The interview guide addresses three primary topics and collects participants’ demographics. First, it asks participants to describe their case history, including a discussion of both the initial genetic test results and the reclassification. Second, it explores participants’ comprehension of these results. Finally, it captures the psychosocial impact of the reclassification on the participant.

### 2.3 Data analysis

Phone‐recorded interviews were conducted by an experienced interviewer trained in qualitative methods (CMEH) using the interview guide. Recruitment continued until thematic saturation was reached. All interviews were professionally transcribed verbatim and de‐identified. Each participant was given a pseudonym, consisting of a randomly assigned number and an annotation of the original and reclassified category of their result (P = pathogenic, LP = likely pathogenic, U = VUS, LB = likely benign, B = benign). Grounded theory was used to analyze the interviews in order to ascertain common themes across participants’ experiences (Strauss & Corbin, 1998). Two authors (CMEH, BCW) developed a code list after coding an initial subset of transcripts. All authors deliberated over the themes and patterns that emerged from the interviews, and coding was refined through an iterative process. Two authors (CMEH, BCW) then coded all transcripts. Conflicts in interpretation were discussed with the whole team and resolved by consensus.

## 3 RESULTS

### 3.1 Respondents

Initially, 41 individuals met enrollment criteria. Twenty of these candidates consented and completed an interview, resulting in a total response rate of 49%. Participants had been notified of the reclassification between two and six years prior to the interview, with an average of four years having passed. The demographics of the study population are fairly representative of the patient population in the clinic who had a result reclassified. Only one man (5%) completed the interview, while men make up the minority in the patient population as well (15%). The cohort included six African Americans (30%; 7% of the patient population) and 14 Caucasians (70%; 75% of the patient population). Ages ranged from 35 to 70, with an average age of 51. Six of the participants (30%) had had reclassifications that, according to current recommendations, warranted a change in surveillance or treatment (Turner et al., 2019). The plurality (9, or 45%) had a genetic test result associated with an increased risk for developing breast cancer. (These final data points are not available for the broader patient population.) For more demographic information, see Table 1.

**Table 1:** Participant characteristics

Participant characteristics		*N* (%)
Gender	Female	19 (95%)
	Male	1 (5%)
Ethnicity	African American	6 (30%)
	Caucasian	14 (70%)
Age category	30–39	4 (20%)
	40–49	5 (25%)
	50–59	7 (35%)
	60–69	3 (15%)
	70–79	1 (5%)
Educational attainment	High school or less	1 (5%)
	Some college	8 (40%)
	College	2 (10%)
	Graduate	9 (45%)
Clinical concern^a^	Breast cancer	9 (45%)
	Lynch syndrome	3 (15%)
	Other	8 (40%)
Direction of reclassification	Upgrade (VUS > LP/P, LP > P)	10 (50%)
	Downgrade (P > LP/VUS, VUS > LB/B)	10 (50%)

^a^ Some patients had multiple reclassifications.

### 3.2 Means of recontact

Our participants were evaluated at the Hereditary Cancer Clinics and received pre‐ and post‐test genetic counseling. They were sent personal notification of any reclassification of their genetic test results. We confirmed that all 20 participants with whom we interviewed had received written or oral communication with this reclassification information. Nine of the interviewees had also directly communicated with a clinician either over the phone or in clinic about the reclassification.

Participants were first made aware of their reclassification in a number of different ways, following clinic policy for recontact depending on whether the variant was up‐ or downgraded. Surprisingly, some of the participants recalled being contacted directly by the laboratory; others did not recall the reclassification. In some instances, the genetics specialist called the patient before sending the updated genetic test report. In other instances, participants received only a letter by mail or a message through the online patient portal.

One participant in her 30s learned of the reclassification through a family member. She had initially undergone genetic testing because of her strong family history of breast and ovarian cancers and was told that she had a pathogenic *BRCA2* mutation. Because of the increased lifetime risk for developing breast cancer, her surgeon recommended a double mastectomy and reconstruction. Shortly before her scheduled surgery, her niece underwent genetic testing at a different clinic, using a different laboratory. The niece had the same *BRCA2* variant, but her laboratory categorized it as a VUS, therefore making it clinically insufficient to warrant the invasive prophylactic surgical interventions. Confused by this discrepancy, the participant contacted her genetic clinician, who had not been informed about the change in interpretation, which had occurred in the time between the participant's and her niece's tests. “It makes me feel unnerved,” she explained (03P > U). Because the laboratory used for this participant had not notified the clinic of the reclassification, the only way she could have learned of it was through her niece's serendipitous report. Had both relatives been tested by the first laboratory, the participant suspects that she and her niece would have undergone unnecessary surgery.

### 3.3 Psychosocial impact

Few participants expressed disappointment, worry, lack of trust, or frustration related to their reclassification. Several interviewees had changed surveillance behaviors, which they considered minor, or had begun to pursue a prophylactic surgery but had changed their mind or had received the reclassification before they had undergone the intervention. These interviewees did not describe the reclassification as disturbing nor did they consider it a medical error.

Only one participant reported undergoing an irreversible medical intervention based on a genetic test result that was later downgraded. A participant with breast cancer in her fifties was initially told that she had a likely pathogenic genetic test result in the *TP53* gene related to Li‐Fraumeni Syndrome. Bilateral mastectomy was recommended by her surgeons, “and I acted on that with the knowledge of the Li‐Fraumeni diagnosis” (24LP > U), she told us. She was called into clinic the following year, at which point she was informed that her *TP53* genetic test result had been reinterpreted as a VUS, and that prophylactic surgeries were no longer recommended. She perceived that she was expected to be relieved because Li‐Fraumeni is often considered to be a devastating diagnosis, but instead she and her husband “were pretty angry. […] It's easy to jump to ‘This was quite a mistake!’” However, she refused “living in a place of anger and resentment” and instead said that she respects the laboratory for its reclassification efforts and characterized her current feelings – now two years after the surgery – as ones of increased caution but not decreased trust. “[The reclassification] just highlighted to me how much is not known. […] I have a lot of respect for the field of medicine, [but] we can identify more than we can understand.”

The overall impact of reclassification on our participants’ relationship with the institution or medicine in general was limited. Only three (15%) participants said that the reclassification decreased their confidence in medical genetics. Furthermore, only two (10%) said that they were less confident in the accuracy of their test result's interpretation at the time of the interview, and four (20%) said that they believed the result's interpretation might or would likely change in the future.

### 3.4 Participants often recalled little about reclassifications

While all respondents recalled their original test results, most interviewees appeared to focus limited attention on the new interpretation or its communication, often recalling little about the experience or the new information. A participant in her late sixties, who had a VUS downgraded to benign, described her reclassification as follows: “I’m not saying it was even much of a change or *any* [kind] of a change. As I read it, it was almost like it didn't really pertain to me. […] I couldn't even tell you what it is” (10U > B). A woman in her mid‐sixties, who had a result reclassified from likely pathogenic to pathogenic said of the updated report, “there's probably some fine print here, but [I’m] not seeing the difference, so whatever. […] To me it was, ‘We used to call it Gobbledygook, but now we're calling it Gobbledygook Part B’” (23LP > P).

Participants without clinically meaningful reclassifications were not the only ones who disregarded the new information they received. Six (43%) of the participants with clinically meaningful reclassifications that could potentially alter management, including VUSs downgraded to benign or likely benign, also expressed little interest in or understanding of the updated interpretation. Moreover, many participants had little or no memory that a reclassification had occurred and been reported to them. “I’m sure I did” get a reclassification, a woman with a clinically meaningful downgrade related to breast cancer told us (03P > U). “Maybe. I can't remember. Oh man, this is a whirlwind.” Three participants completely denied that their genetic test result had been reclassified. All three of these participants were notified of their reclassification by letter.

Less often, participants did remember receiving their results. Some of these individuals even had concrete understandings of the reinterpretation's implications for their health care. A woman diagnosed with pheochromocytoma remembered that she had initially been given “inconclusive” results that had been reclassified as “a recognized mutation,” in her own words (27U > LP). She believed the updated information was “beneficial” for herself and her family.

## 4 DISCUSSION

### 4.1 Overall results

In order for genetic medicine to be successfully mainstreamed, patients and clinicians must understand and appreciate the uncertainties inherent in the practice (Haga et al., 2013;Syurina, Brankovic, Probst‐Hensch, & Brand, 2011). However, this area has been understudied, particularly regarding patients’ responses to the reclassification of their genetic test results. In this article – the first to focus on patients’ experiences with the reclassification of clinical genetic results – we describe many instances in which patients did not understand or recall the reclassification but did not necessarily experience distress.

We entered this project anticipating that participants with reclassifications – especially clinically meaningful reclassifications in either direction – would have increased worries about or frustrations with genetic testing. We hypothesized that the notification of reclassification might decrease patients’ trust in medical genetics and that patients might have perceived the initial interpretation to be a medical error. Despite much confusion and misunderstanding, however, our interviewees by and large did not experience these anticipated negative psychosocial outcomes. In fact, when participants remembered that they had received an updated report, they were typically pleased, feeling that the notification demonstrated their care team's continued attention and concern about their well‐being.

Despite having received notification of the reclassification – often through multiple modalities – many participants neither remembered nor appreciated the significance of the reclassification for their health care. It is not necessarily unanticipated that patients lack a robust recall of genetic test results (McCullum et al., 2007;Skytte et al., 2010;Vos et al., 2008), and we have previously shown that patients, especially patients with hereditary cancers, are often overwhelmed by numerous tests, making it difficult for their already stretched emotional and energetic resources to attend to all relevant new information about their health and health care (Halverson, Wessinger, Clayton, & Wiesner, 2019). However, this is the first time such an issue has been demonstrated regarding reclassifications. A small percentage of our participants were able to articulate a concrete understanding of their reclassification's meaning and its consequences on their trust in medical genetics. Overall, however, the reclassifications appeared to leave little impression on our interviewees.

This is particularly concerning as reclassification is becoming increasingly common. A recent study found that over 7% of over 1,600 hereditary cancer test reports had had a genetic variant reclassified within five years of its initial report (Turner et al., 2019). Garcia and colleagues reported that 56% of women with VUSs in *BRCA* genes received reclassifications over a similar period (Garcia et al., 2014). Other studies have reported frequencies of reclassification that vary based on disease type, gene, and ancestry, among other factors (Baudhuin, Kluge, Kotzer, & Lagerstedt, 2019;Dong et al., 2019;Park et al., 2017;SoRelle, Thodeson, Arnold, Gotway, & Park, 2019;Wright et al., 2018).

Studies have shown that patients and clinicians have difficulty understanding inconclusive genetic test results, a category that includes reinterpretations (McCullum et al., 2007;Skytte et al., 2010;Vos et al., 2008). It can be challenging for both patients and clinicians to navigate the nuanced and evolving clinical relevance of genetic test results, as illustrated by the interview of our participant with the downgraded *TP53* variant. This individual's case provides a cautionary tale, as irreversible surgeries were performed using data that was, at the time, the best available to the patient and surgeon. Moreover, just because the variant was downgraded does not mean that it is benign.

Another lesson learned from this study relates to the role of laboratories in reanalyzing variants and returning updated interpretations. Indeed, there is no consensus regarding how frequently clinical genetic results should be reexamined or how changes should be reported (Carrieri et al., 2017;David et al., 2019), although many argue that genetic tests should be reexamined periodically and new interpretations returned in both clinical and research settings (Bombard et al., 2019;Bredenoord, Onland‐Moret, & Van Delden, 2011;Carrieri et al., 2017). While expert clinicians may choose to reexamine the impact of particular variants themselves, most reinterpretation is undertaken by laboratories, which vary in whether and how they communicate new analyses (Chisholm et al., 2018).

The case of the participant and niece with a downgraded *BRCA2* variant demonstrates the danger that the lack of harmonization of classification schemes across laboratories represents. Currently, many laboratories that conduct research that can lead to reclassifications neither routinely reanalyze old patient cases nor return these results (Green et al., 2013). This means that data about patients’ experience with reclassifications are strikingly difficult to find. This dearth of data is worrying both ethically and legally. The failure to notify a patient of a reclassification has already been the grounds for one lawsuit (Seymour, Case y, & Moylan, 2018), and we describe one participant who only learned about the reclassification from a relative who had been tested in another center. Inconsistent variant calling is a major hurdle for mainstreaming medical genetics, which may well be compounded with the increasing use of genetic tests for diagnosis and predictive healthcare (Balmaña et al., 2016;Slavin, Blazer, & Weitzel, 2016). As the science of genetics evolves, it will become increasingly important for laboratories to be aware of differing classifications by competitors and to harmonize classifications accordingly. It will also be important for patients, their families, and their genetics providers to keep an open line of communication.

### 4.2 Limitations of the current study

One limitation of our study is that the perceptions are based on participant recall and not on the actual counseling process. Another is that the initial testing and the reclassification notification took place several months to several years before our interviews. While the delay might have increased the likelihood that participants did not remember or appreciate the reinterpretation of their results, it does reflect the reality of clinical genetics, where there are long gaps between testing and potential reclassification. That said, all participants remembered at least the initial testing and return of results, which suggests that for many the reinterpretation was less important.

## 5 CONCLUSION

The reinterpretation of a given variant can in principle have a significant impact on the management of a patient's care as well as that of their family members. This is the first study that provides a real‐world snapshot of patients’ perceptions about the reclassification of variants related to cancer susceptibility. Our participants experienced minimal harm in learning about their reclassifications. While some respondents clearly understood the new interpretations, many of our interviewees expressed confusion or lack of recall about their reclassifications. Understanding patients' responses to reclassification is therefore necessary to determine how best to return these results in a meaningful and ethical way.

## AUTHOR CONTRIBUTIONS

CMEH and GLW conceived of the presented idea. CMEH and BCW collected the data. All authors – CMEH, LMC, BCW, GLW, and EWC – performed the analysis, drafted the manuscript, and edited the final manuscript.

## Data Availability

The authors confirm that the data supporting the findings of this study are available within the article's supplementary materials.
